# BMI Change During Puberty Is an Important Determinant of Adult Type 2 Diabetes Risk in Men

**DOI:** 10.1210/jc.2018-01339

**Published:** 2018-12-04

**Authors:** Claes Ohlsson, Maria Bygdell, Maria Nethander, Annika Rosengren, Jenny M Kindblom

**Affiliations:** 1 Centre for Bone and Arthritis Research, Institute of Medicine, Sahlgrenska Academy at University of Gothenburg, Gothenburg, Sweden; 2 Bioinformatics Core Facility, Sahlgrenska Academy at University of Gothenburg, Gothenburg, Sweden; 3 Department of Molecular and Clinical Medicine, Sahlgrenska Academy at University of Gothenburg, Gothenburg, Sweden

## Abstract

**Objective:**

The aim of this study was to determine the role of change in body mass index (BMI) during puberty, independent of childhood overweight, for risk of adult type 2 diabetes in men.

**Study Design, Population, and Outcome:**

We included 36,176 men who had weight and height measured at age 8 and 20 years available from the BMI Epidemiology Study and the Conscription register. Information on type 2 diabetes (n = 1,777) was retrieved from the Swedish National Patient Register. Hazard ratios and 95% CIs were estimated by Cox regressions including birth year and country of birth as covariates. Because the assumption of proportional hazards was violated for the association between BMI change during puberty and type 2 diabetes, we split the follow-up time into early (≤55.7 years) and late (>55.7 years).

**Results:**

Both childhood overweight and high BMI increase during puberty associated with risk of adult type 2 diabetes. Men with childhood overweight that normalized during puberty did not have significantly increased risk of type 2 diabetes [early type 2 diabetes 1.28 (0.89; 1.82); late type 2 diabetes 1.35 (0.97; 1.87)]. Men who developed overweight during puberty [early 4.67 (3.90; 5.58); late 2.85 (2.25; 3.61)] and men overweight throughout childhood and puberty [early 4.82 (3.84; 6.05); late 3.04 (2.27; 4.06)] had substantially increased risk of type 2 diabetes compared with men who were never overweight.

**Conclusion:**

BMI change during puberty is an important and childhood BMI a modest independent determinant of adult type 2 diabetes risk in men.

## Teaser

Both overweight at 8 years and a high BMI increase during puberty were associated with increased risk of adult type 2 diabetes in the BMI Epidemiology Study (n = 36,176; cases of type 2 diabetes, n = 1,777).

The incidence of type 2 diabetes is rising, coinciding with the global obesity epidemic observed among both adults and children during the last three decades (1–3). The causal association between a high adult body mass index (BMI) and increased risk of type 2 diabetes is well established, and prevention of obesity remains among the most important measures to reduce the burden of type 2 diabetes (4).

Recent studies provide strong evidence for an association between a high BMI at young adult age and risk of type 2 diabetes later in life (5, 6), and some studies reported that high childhood BMI (7–14) and low birth weight (15) are associated with increased risk of adult type 2 diabetes. Childhood and puberty represent two physiologically distinct developmental periods. As previously reported, the correlation between childhood BMI and BMI change during puberty in the BMI Epidemiology Study Gothenburg is only marginal (16), and this correlation has not been evaluated in previous studies. In contrast, the correlation between BMI at 8 and 20 years of age is high in the present cohort (*r* = 0.61), in accordance with other studies such as the Copenhagen School Health Record Register (BMI at age 7 and BMI at age 18 to 19, *r* = 0.55) (3) and the Young Finns study including men born 1962 to 1977 (BMI at 9 and BMI at 36 years, *r* = 0.54) (4). Thus, childhood BMI and BMI change during puberty have the potential to contribute nonoverlapping information as risk markers for adult disease (16). This notion is supported by our recent findings that excessive BMI increase during puberty is an independent risk marker of cardiovascular disease mortality (16), stroke (17), and heart failure (18) in men. The independent role of BMI change during the pubertal period for adult type 2 diabetes risk in men is unknown.

In a study from four populations in which BMI was measured both in childhood at 3 to 19 years of age and later in the same participants at 30 to 40 years of age, overweight adults had increased risk of adult type 2 diabetes regardless of BMI status during childhood (10). The risk in overweight children was similar to the risk in participants who were never overweight, but this analysis was underpowered including only two overweight boys and one overweight girl who developed adult type 2 diabetes. In addition, it was not possible to determine the association specifically for BMI change during puberty because the childhood BMI measurements for a majority of the participants were not done before onset of puberty but rather during the course of puberty, and the adult BMI measurements were done up to about 25 years after cessation of puberty (10).

The main aim of the current study was to determine the independent role of BMI change during puberty for the risk of adult type 2 diabetes in men, which requires BMI measurements both before and after puberty. To this end, we used the population-based BMI Epidemiology Study (BEST) in Gothenburg, Sweden, with information on both prepubertal childhood BMI and young adult BMI shortly after puberty in men. As BMI change during puberty is an independent risk marker of visceral adiposity (19) and cardiovascular mortality (16) in the BEST cohort, we hypothesized that it might also be a risk marker of adult type 2 diabetes in men.

## Methods

### Study design and participants

The BMI Epidemiology Study Gothenburg (BEST Gothenburg) was initiated with the overall aim to study the impact of birth weight, childhood, and adolescent BMI on adult diseases. To that end, we collected data on birth weight as well as directly measured height and weight from centrally archived School Health Care (SHC) records for all men born 1945 to 1961 in Gothenburg, Sweden. We also collected height and weight at young adult age from military conscription tests, kept by the Swedish Defense Recruitment Agency. Data from conscription (young adult age) have been added to the database for the individuals with an SHC record in the central archive and a complete Personal Identity Number (PIN). Conscription was mandatory until 2008 for all Swedish men.

For ∼100 years, the general health and well-being of Swedish children have been followed by SHC from school start (at age 7 for the study cohort) throughout the school years. Sweden has had a compulsory school attendance since 1936, starting from the year the child turned 7. The SHC program includes vaccinations and direct measurements of height and weight performed by specially trained school nurses. From the early 1950s (coinciding with childhood measurements from birth year ≈1943), 98.5% of all pupils nationwide were covered by SHC (20).

**Figure 1.**
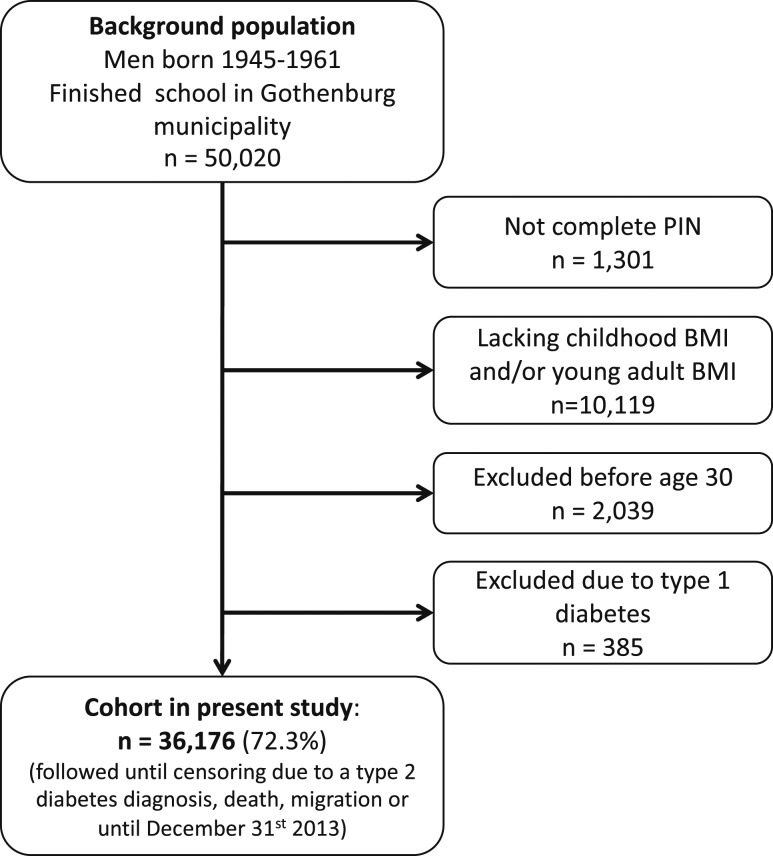
Flow chart of included individuals.

The study cohort was linked to high-quality national disease registers using the PINs from the included subjects. Eligible individuals were those with an SHC record in the central archive and a 10-digit PIN (Fig. 1). Subjects with data available for calculation of both childhood BMI and young adult BMI were included in the current study (Fig. 1; Table 1). The following subjects were excluded before study start: 1) subjects with incomplete PIN, 2) subjects lacking childhood BMI or young adult BMI, 3) subjects who died, emigrated, or were diagnosed with diabetes before 30 years of age, and 4) subjects who were diagnosed with type 1 diabetes at any time point (in total, 27.7% excluded; Fig. 1). The 36,176 men included in the study were followed from 30 years of age until censoring due to a type 2 diabetes diagnosis (n = 1,777), migration (n = 1,900), death (n = 2,756), or until 31 December 2013, whichever came first. Subjects who were censored during the study (*i.e.*, those who emigrated, died, or were diagnosed with type 2 diabetes) were included in the study until the date of censoring.

**Table 1.** Cohort Description

	Mean (SD)
Exposures	
Childhood BMI (8 y), kg/m^2^	15.7 (1.4)
Young adult BMI (20 y), kg/m^2^	21.4 (2.5)
ΔpBMI (20 − 8 y), kg/m^2^	5.6 (2.0)
Birth weight, kg^*a*^	3.6 (0.6)
Childhood overweight, n (%)	2,256 (6.2)
Childhood obesity, n (%)	486 (1.3)
BMI Z-score, WHO reference (8 y)	−0.005 (0.9)
Young adult overweight, n (%)	2,665 (7.4)
Young adult obesity, n (%)	306 (0.8)
Country of birth: Sweden, n (%)	30,370 (84.0)
Other, n (%)	5,806 (16.0)
Outcomes	
Type 2 diabetes after 30 y of age	1,777
Mean (SD) age at type 2 diabetes diagnosis	55.0 (7.0)
Median (IQR) age at type 2 diabetes diagnosis	55.7 (50.9–60.1)

Total cohort, n = 36,176. Childhood overweight and obesity at 8 y of age is defined as BMI ≥17.9 kg/m^2^ and 20.0 kg/m^2^, respectively, whereas young adult overweight and obesity at 20 y of age is defined as BMI ≥25 kg/m^2^ or 30 kg/m^2^, respectively.

Abbreviations: ΔpBMI, BMI change during puberty; IQR, interquartile range; WHO, World Health Organization.

^a^ Birth weight was available in a subsample (n = 34,265).

The ethics committee of the University of Gothenburg, Sweden, approved the study and waived the requirement for written informed consent. There was no commercial sponsorship.

### Cohort representativeness

We have previously evaluated the representativeness of the cohort by comparison with the conscription register and found that young adult BMI at conscription was similar for the included and the not included subjects (16), suggesting that the cohort is representative for Gothenburg, the second largest city in Sweden.

### Definition of study exposures

Prepubertal childhood BMI at 8 years of age and young adult BMI at 20 years of age were calculated using all paired height and weight measurements in the period between 6.5 and 9.5 years of age for prepubertal childhood BMI, and in the period 17.5 to 22 years of age for young adult BMI. All measurements within these intervals were used to construct a linear regression, and the data for individual subjects were then adjusted (extrapolated or intrapolated) on this regression to obtain BMI at 8 and 20 years of age. This age-adjusted 8-year BMI/20-year BMI was used to classify subjects as overweight or obese at 8 years of age [using the Centers for Disease Control and Prevention cutoffs at 8 years of age for overweight: BMI ≥17.9 kg/m^2^, and for obesity: BMI ≥20.0 kg/m^2^ (21)] and at 20 years of age (based on BMI >25 or 30 kg/m^2^, respectively). Overweight refers to the population with a BMI above the overweight cutoff and includes both overweight and obese subjects at either 8 or 20 years of age, respectively. BMI change during puberty was defined as the difference between young adult BMI and childhood BMI. Onset and duration of puberty vary between individuals, and pubertal development greatly alters body composition (22). To avoid the confounding effect of ongoing puberty on BMI, we defined the pubertal period with a rather wide time window. Therefore, the time between 8 and 20 years included not only the complete pubertal period but also periods of varying length both before and after puberty. Birth weight was retrieved from SHC records. Gestational age was not available.

Information on country of birth and migration was retrieved from the Longitudinal Integration Database for Health Insurance and Labor Market Studies held at Statistics Sweden. Country of birth was categorized as Sweden (the study subject and both his parents born in Sweden) or not Sweden (the subject or one or both parents not born in Sweden or information on country of birth was missing). Country of birth was included as a covariate because associations between BMI and risk of diabetes have been shown to be significantly modified by ethnicity (23).

### Definition of study outcomes

Linkage to registers held by the National Board of Health and Welfare and Statistics Sweden was performed using the individuals’ unique 10-digit PIN. Dates and diagnoses for the first appearance of a diagnosis of type 2 diabetes in any coding position were retrieved from the National Patient Register, initiated in 1964 and with full coverage in the Gothenburg region from 1972. A diagnosis of type 2 diabetes was defined according to the *International Classification of Diseases* (ICD) system codes: E11 in ICD10 and 250 occurring for the first time after 30 years of age in ICD8 and ICD9.

### Statistical analysis

There were no missing values for the main parameters (childhood BMI, BMI change during puberty, young adult BMI, country of birth, birth year, or follow-up). The only parameter without a complete set of data were birth weight (n = 34,265, which corresponds to 95% of the entire cohort). Models including birth weight only included the subgroup of boys with birth weight available. Childhood BMI, young adult BMI, and birth weight were log-transformed and standardized, and BMI change during puberty was standardized when used in the Cox regression models. The standardization was done within the study population, having zero mean and unit variance. The standard score (Z) was calculated as Z = (x − *μ*)/*σ*. *μ* is the mean of the study population, and *σ* is the SD of the study population. We used Cox proportional hazards regression to analyze the association between exposures and events. Nonlinear associations were evaluated by inclusion of a quadratic term and if significant, further evaluations were undertaken using a restricted cubic spline-approach in the Cox regression analysis for a flexible nonlinear assessment of the hazard ratio (HR) in relation to childhood BMI (24). Three knots placed at the childhood BMI percentiles 5, 75, and 95 and 5, 85, and 95, respectively, were found to give a small Akaike Information Criterion and capture the average curve shape over a systematic assessment of different alternatives. Kaplan-Meier survival plots were performed with study subjects divided according to overweight status at childhood and young adult age and significance tested using log-rank test between the groups. The assumption of proportionality in the Cox regression models was assessed both through visual evaluations of Schoenfeld residual plots and through proportional hazard tests using the “survival” package in the R statistical software. A violation of the assumption of proportional hazards was detected for BMI change during puberty, but not for childhood BMI, birth weight, birth year, or country of birth. We therefore split the follow-up at the age of the median case of type 2 diabetes (55.7 years). Kaplan-Meier survival plots, analyses using restricted cubic splines, and the test for proportionality were done in R using the “survival” and “rms” packages (25–27), and all other analyses were performed in SPSS Statistics 25.

## Results

### Study cohort

In this population-based study, 36,176 men (>72% of the background population) born 1945 to 1961 with information on both childhood BMI at age 8 and BMI change during puberty (BMI at age 20 − BMI at age 8) were included and followed until December 2013 (Fig. 1; Table 1). Mean follow-up starting from 30 years of age was 28.3 years (1,025,253 person-years of follow-up). There were 1,777 cases of type 2 diabetes before the end of follow-up, and the median age at diagnosis was 55.7 years (Table 1).

### Childhood overweight and BMI change during puberty and the association with risk of adult type 2 diabetes

**Figure 2.**
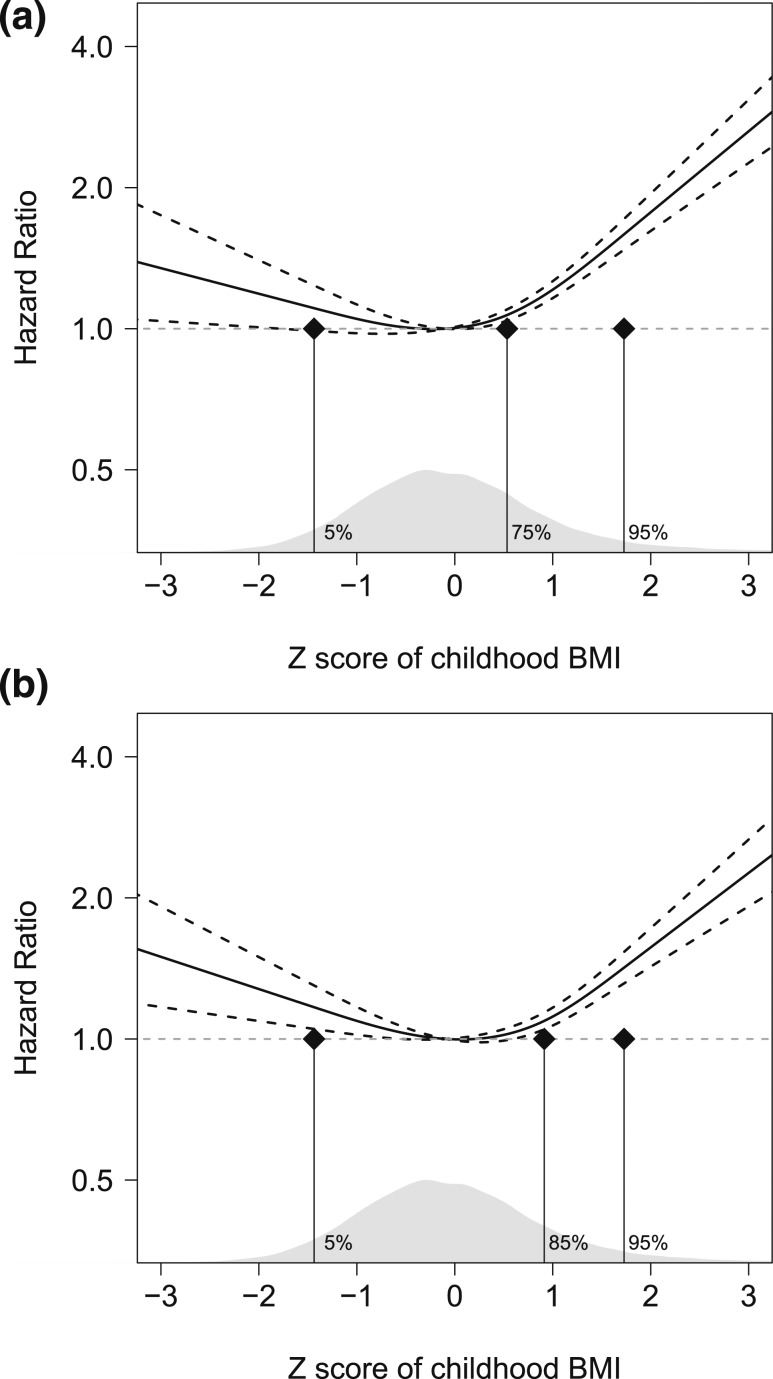
Smoothed plots of HRs for type 2 diabetes according to childhood BMI at 8 y of age in 36,176 Swedish men. Cox regression analysis using a restricted cubic spline approach for a flexible nonlinear assessment of the HR for risk of type 2 diabetes in relation to (a) childhood BMI at 8 y of age (*P* < 0.001 for nonlinearity) and (b) childhood BMI at 8 y of age adjusted for BMI change during puberty (*P* < 0.001 for nonlinearity), after 30 y of age. The figure shows HR (*y*-axis) and Z-score of childhood BMI (*x*-axis). Three knots were placed at the childhood BMI percentiles (a) 5, 75, and 95 and (b) 5, 85, and 95, respectively (indicated by vertical black lines). The models were adjusted for birth year and country of birth. Data are presented as HR (black line) ± 95% CI (black dashed line). The distribution of subjects according to childhood BMI is shown in gray in the bottom part of the figure. The horizontal dashed line corresponds to the reference (mean childhood BMI 15.7 kg/m^2^) HR of 1.0 (no excess rate of events).

Cox regression models adjusted for birth year and country of birth revealed that a high childhood BMI was associated with increased risk of type 2 diabetes in a nonlinear manner (inclusion of cBMI^2^, *P* < 0.05). Restricted cubic spline analyses confirmed a statistically significant nonlinear association between childhood BMI and type 2 diabetes [Fig. 2(a)] that remained after adjustment for BMI change during puberty [Fig. 2(b)]. The increased risk of type 2 diabetes was mainly observed in subjects that were overweight at childhood compared with normal weight children [HR 2.09 (1.80; 2.41)], and this association remained significant after adjustments for BMI change during puberty [HR 1.77 (1.53; 2.06)]. Moreover, the restricted cubic splines analysis indicated that a very low BMI at 8 years of age was associated with increased risk of type 2 diabetes [Fig. 2(b)]. To explore this further, we analyzed the risk for type 2 diabetes for a similar part of the cohort as those with childhood overweight [*i.e.*, the 6.2% (n = 2,256) with the lowest BMI at age 8] vs those with normal weight. This analysis, adjusted for BMI change during puberty, showed an increased risk for those with very low childhood BMI [HR 1.32 (1.02; 1.72)] compared with those with a normal BMI.

When evaluating the association between BMI change during puberty and the risk of type 2 diabetes, we observed violations of the assumption of proportional hazards indicating that the strength of the association declined by follow-up time. We therefore split the follow-up at the median age of the cases of type 2 diabetes (55.7 years) and present the associations for both early (≤55.7 years) and late (>55.7 years) type 2 diabetes. A high BMI increase during puberty was linearly associated with an increased risk of both early [HR per SD increase 1.66 (1.59; 1.73)] and late [HR 1.42 (1.34; 1.50)] type 2 diabetes, and these associations remained significant after adjustment for childhood BMI as well as for young adult BMI (Table 2). Thus, both childhood overweight and a high BMI increase during puberty are associated with increased risk of adult type 2 diabetes, particularly early type 2 diabetes.

**Table 2.** HRs for Type 2 Diabetes in Relation to BMI Change During Puberty in 36,176 Swedish Men Followed for a Mean of 28.3 (6.9) Years After 30

	Early Type 2 Diabetes HR per SD (95% CI)	Late Type 2 Diabetes HR per SD (95% CI)
Base model	1.66 (1.59; 1.73)	1.42 (1.34; 1.50)
Adjusted model 1	1.61 (1.54; 1.68)	1.39 (1.32; 1.47)
Adjusted model 2	1.29 (1.17; 1.42)	1.25 (1.11; 1.40)

HRs for type 2 diabetes for BMI change during puberty were calculated using Cox proportional hazards regression. The base model is adjusted for birth year and country of birth, and the adjusted models are additionally adjusted for either childhood BMI at 8 y of age (adjusted model 1) or young adult BMI at 20 y of age (adjusted model 2). Number of early (≤55.7 y of age) type 2 diabetes diagnoses, n = 889; total, n = 36,176; number of late (>55.7 y) type 2 diabetes diagnoses, n = 888; total, n = 26,015 [the 36,176 minus those censored before 55.7 y of age due to 1) type 2 diabetes diagnosis (n = 889); 2) death (n = 1,860); 3) migration (n = 1,739), or 4) not yet reached the age of 55.7 y on 31 December 2013 (n = 5,673)]. The total number of censored subjects in the analysis of late type 2 diabetes, n = 10,161. *P* ≤ 0.001 for all analyses. The analyses in Table 2 have been performed with stratified follow-up due to fact that the assumption of proportional hazards was not fulfilled. *χ*^2^ goodness-of-fit tests were performed for each of the adjusted models against the base model for both early and late type 2 diabetes. The goodness-of-fit tests revealed that both childhood BMI (adjusted model 1) and adult BMI (adjusted model 2) add extra information to the base model (*P* < 0.001 for both early and late type 2 diabetes).

### Risk of adult type 2 diabetes in relation to overweight status at childhood and at young adult age

Overweight was observed in 6.2% and 7.4% of the subjects at childhood and at young adult age, respectively, and as many as 58% of the subjects with childhood overweight became normal weight at young adult age, whereas 64% of those who were overweight at young adult age had been normal weight at 8 years of age and had developed their overweight during the pubertal period (Table 3). We next evaluated the impact of change in overweight status during the pubertal period for risk of adult type 2 diabetes. Men with childhood overweight that normalized during puberty did not have a significantly increased risk of type 2 diabetes (early HR 1.28 [0.89; 1.82]; late HR 1.35 [0.97;1.87]), whereas men who developed overweight during puberty had a substantially increased risk of type 2 diabetes (early HR 4.67 [3.90; 5.58]; late HR 2.85 [2.25; 3.61]), reaching similar increased risk as observed in men overweight at both childhood and young adult age (early HR 4.82 [3.84; 6.05]; late HR 3.04 [2.27; 4.06]) compared with men who were never overweight (Table 3). A similar pattern was observed when obesity at childhood and young adult age was evaluated (Table 4).

**Table 3.** Risk of Adult Type 2 Diabetes in Relation to Overweight Status at Childhood and at Young Adult Age

Childhood/Young Adult Overweight Status	Type 2 Diabetes	HR (95% CI)
Early type 2 diabetes (≤55.7 y of age; n = 36,176)		
Normal weight/normal weight (n = 32,202)	620	1 (reference)
Overweight/normal weight (n = 1,309)	32	1.28 (0.89; 1.82)
Normal weight/overweight (n = 1,718)	152	4.67 (3.90; 5.58)
Overweight/overweight (n = 947)	85	4.82 (3.84; 6.05)
Late type 2 diabetes (>55.7 y of age; n = 26,015)		
Normal weight/normal weight (n = 23,441)	726	1 (reference)
Overweight/normal weight (n = 922)	37	1.35 (0.97; 1.87)
Normal weight/overweight (n = 1,044)	76	2.85 (2.25; 3.61)
Overweight/overweight (n = 608)	49	3.04 (2.27; 4.06)

HRs for type 2 diabetes were calculated using Cox proportional hazards regression. Normal weight/normal weight, not overweight at 8 or 20 y of age; Overweight/normal weight, overweight at 8 but not at 20 y of age; Normal weight/overweight, overweight at 20 but not at 8 y of age; Overweight/overweight, overweight both at 8 and 20 y of age. Childhood overweight at 8 y of age was defined as BMI ≥17.9 kg/m^2^ (21), whereas young adult overweight at 20 y of age was defined as BMI ≥25 kg/m^2^. The models have been adjusted for birth year and country of birth.

**Table 4.** Risk of Adult Type 2 Diabetes in Relation to Obesity Status at Childhood and at Young Adult Age

Childhood/Young Adult Obesity Status	Type 2 Diabetes	HR (95% CI)
Early type 2 diabetes (≤55.7 y of age; n = 36,176)		
Normal weight/normal weight (n = 35,489)	808	1 (reference)
Obesity/normal weight (n = 381)	24	2.78 (1.85; 4.17)
Normal weight/obesity (n = 201)	36	8.19 (5.86; 11.45)
Obesity/obesity (n = 105)	21	10.59 (6.86; 16.33)
Late type 2 diabetes (>55.7 y of age; n = 26,015)		
Normal weight/normal weight (n = 25,597)	853	1 (reference)
Obesity/normal weight (n = 257)	16	2.18 (1.33; 3.58)
Normal weight/obesity (n = 109)	14	5.38 (3.17; 9.12)
Obesity/obesity (n = 52)	5	3.51 (1.46; 8.45)

HRs for type 2 diabetes were calculated using Cox proportional hazards regression. Normal weight/normal weight, no obesity at 8 or 20 y of age; Obesity/normal weight, obesity at 8 but not at 20 y of age; Normal weight/obesity, obesity at 20 but not at 8 y of age; Obesity/obesity, obesity both at 8 and 20 y of age. Childhood obesity at 8 y of age was defined as BMI ≥20.0 kg/m^2^ (6), whereas young adult obesity at 20 y of age was defined as BMI ≥30 kg/m^2^. The models have been adjusted for birth year and country of birth.

### Adjustment for birth weight, country of birth, and birth year

In the full model including childhood BMI and BMI change during puberty, birth weight was inversely associated with the risk of both early and late type 2 diabetes [early HR 0.83 per SD increase (0.78; 0.88) and late HR 0.81 (0.76; 0.86)]. Adjustment for birth weight (n = 34,265) did not alter the described associations for overweight status at childhood and at young adult age with adult risk of type 2 diabetes (Table 5). The covariate Sweden as country of birth showed a clearly significant inverse association with early but not late risk of type 2 diabetes [early HR 0.79 (0.67; 0.94) and late HR 0.96 (0.79; 1.17)]. The associations for overweight status at childhood and at young age with adult risk of type 2 diabetes were mainly unchanged in the subpopulation including only boys born in Sweden and with parents born in Sweden (n = 30,370; Table 6). For birth year, no clear independent association with the risk of type 2 diabetes was detected [early HR 1.02 (1.00; 1.03) and late HR 0.98 (0.96; 1.01)].

**Table 5.** Risk of Adult Type 2 Diabetes in Relation to Overweight Status at Childhood and at Young Adult Age After Adjustment for Birth Weight

Childhood/Young Adult Overweight Status	Type 2 Diabetes	HR (95% CI)
Early type 2 diabetes (≤55.7 y of age; n = 34,265)		
Normal weight/normal weight (n = 30,495)	577	1 (reference)
Overweight/normal weight (n = 1,234)	31	1.45 (1.00; 2.08)
Normal weight/overweight (n = 1,635)	144	4.85 (4.04; 5.83)
Overweight/overweight (n = 901)	79	4.97 (3.92; 6.29)
Late type 2 diabetes (>55.7 y of age; n = 24,531)		
Normal weight/normal weight (n = 22.090)	677	1 (reference)
Overweight/normal weight (n = 860)	34	1.48 (1.05; 2.09)
Normal weight/overweight (n = 996)	67	2.75 (2.14; 3.54)
Overweight/overweight (n = 585)	47	3.22 (3.40; 4.34)

HRs for type 2 diabetes were calculated using Cox proportional hazards regression after adjustment for birthweight (n = 34,265). Normal weight/normal weight, not overweight at 8 or 20 y of age; Overweight/normal weight, overweight at 8 but not at 20 y of age; Normal weight/overweight, overweight at 20 but not at 8 y of age; Overweight/overweight, overweight both at 8 and 20 y of age. Childhood overweight at 8 y of age was defined as BMI ≥17.9 kg/m^2^ (6), whereas young adult overweight at 20 y of age was defined as BMI ≥25 kg/m^2^. The models have been adjusted for birth year and country of birth.

**Table 6.** Risk of Adult Type 2 Diabetes in Relation to Overweight Status at Childhood and at Young Adult Age in Men Born in Sweden With Parents Born in Sweden

Childhood/Young Adult Overweight Status	Type 2 Diabetes	HR (95% CI)
Early type 2 diabetes (≤55.7 y of age; n = 30,370)		
Normal weight/normal weight (n = 27,062)	506	1 (reference)
Overweight/normal weight (n = 1,099)	23	1.12 (0.74; 1.70)
Normal weight/overweight (n = 1,421)	117	4.50 (3.68; 5.50)
Overweight/overweight (n = 788)	69	4.82 (3.75; 6.20)
Late type 2 diabetes (>55.7 y of age; n = 22,162)		
Normal weight/normal weight (n = 19,968)	612	1 (reference)
Overweight/normal weight (n = 789)	29	1.26 (0.87; 1.82)
Normal weight/overweight (n = 895)	64	2.79 (2.16; 3.61)
Overweight/overweight (n = 510)	42	3.25 (2.38; 4.45)

HRs for type 2 diabetes were calculated using Cox proportional hazards regression in the subpopulation born in Sweden and with parents born in Sweden (n = 30,370). Normal weight/normal weight, not overweight at 8 or 20 y of age; Overweight/normal weight, overweight at 8 but not at 20 y of age; Normal weight/overweight, overweight at 20 but not at 8 y of age; Overweight/overweight, overweight both at 8 and 20 y of age. Childhood overweight at 8 y of age was defined as BMI ≥17.9 kg/m^2^ (6), whereas young adult overweight at 20 y of age was defined as BMI ≥25 kg/m^2^. The models have been adjusted for birth year.

### Kaplan-Meier plots

**Figure 3.**
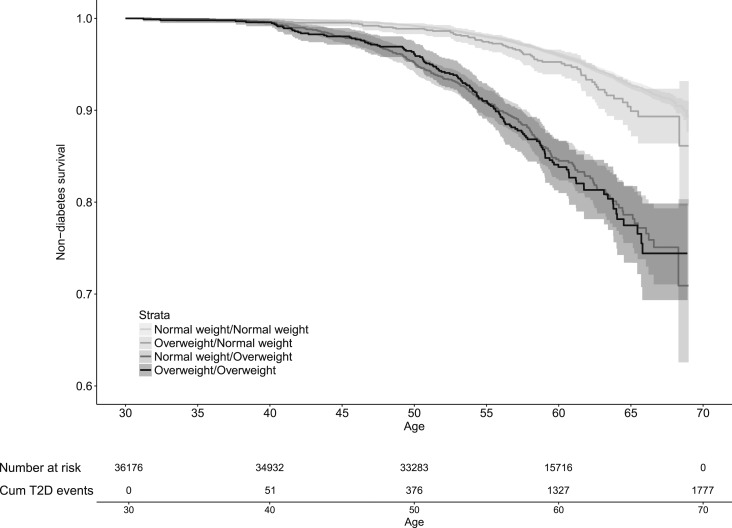
Kaplan-Meier curve of type 2 diabetes (T2D)–free survival according to overweight status at 8 and 20 y of age in 36,176 Swedish men followed for a mean of 28.3 y after age 30. The graph shows type 2 diabetes–free survival according to the following groups of overweight status: Normal weight/Normal weight, not overweight at 8 or 20 y of age; Overweight/Normal weight, overweight at 8 but not at 20 y of age; Normal weight/Overweight, overweight at 20 but not at 8 y of age; and Overweight/Overweight, overweight both at 8 and 20 y of age. Childhood overweight at 8 y of age was defined as BMI ≥17.9 kg/m^2^ (21), whereas young adult overweight at 20 y of age was defined as BMI ≥25 kg/m^2^. The *P* values for comparison between the groups assessed by log-rank and adjusted for multiple testing (three comparisons) were <0.001 for Normal weight/Overweight and Overweight/Overweight vs Normal weight/Normal weight. Cum, cumulative.

Kaplan-Meier survival plots revealed substantially increased risk of type 2 diabetes for men who developed overweight during puberty and in men that were overweight at both childhood and young adult age but not in men with childhood overweight that normalized during puberty compared with men who were never overweight (Fig. 3).

## Discussion

Although it is well established that both a high adult BMI and a high childhood BMI are associated with increased risk of type 2 diabetes, the lack of well-powered studies with BMI measurements both before and after puberty available has made it difficult to determine the independent role of BMI change during puberty for adult risk of type 2 diabetes. We used the BEST cohort including as many as 36,176 individuals and 1,777 cases of type 2 diabetes with data on prepubertal childhood BMI and BMI at young adult age and demonstrated that both childhood overweight and a high BMI increase during puberty associated with increased risk of adult type 2 diabetes. The strength of the latter association was more pronounced for early (≤55.7 years) compared with late-onset (>55.7 years) type 2 diabetes. Men with childhood overweight that normalized during puberty did not have an increased risk of type 2 diabetes, whereas men who developed overweight during puberty had a substantially increased risk of type 2 diabetes, reaching similar increased risk as observed in men overweight at both childhood and young adult age, compared with men who were never overweight. Moreover, subjects with Sweden as country of birth had a lower risk of type 2 diabetes compared with those not born in Sweden or with parents not born in Sweden. However, the associations for childhood overweight and BMI change during puberty with risk of type 2 diabetes are similar in the group with Sweden as country of birth as in the entire cohort.

Given our previous observation that prepubertal childhood BMI and BMI change during puberty correlate only marginally and thus contribute distinct, nonoverlapping information it is crucial to evaluate these two distinct BMI parameters as separate potential risk markers of adult type 2 diabetes (16). Our observation that a high childhood BMI was a modest but independent marker of increased risk of adult type 2 diabetes supports previous studies (7–14). Moreover, in the current study, we were able to identify that the association between childhood BMI and adult risk of type 2 diabetes is nonlinear, mainly driven by subjects who were overweight at 8 years of age.

We also made the observation that a high BMI increase during puberty was robustly associated with an increased risk of type 2 diabetes. This association remained significant after adjustment for childhood BMI and young adult BMI, suggesting that BMI change during puberty confers unique information in the prediction of adult type 2 diabetes. Overweight status changed substantially during the pubertal period in the study cohort. As many as 58% of the subjects with childhood overweight transitioned to normal weight at young adult age, and 64% of the subjects with overweight at young adult age developed their overweight during the pubertal period in the BEST Gothenburg cohort. Using this information, we were able to determine the impact of change in overweight status between childhood and young adult age for risk of adult type 2 diabetes. Of note, men with childhood overweight that normalized during puberty did not have an increased risk of type 2 diabetes, whereas men with overweight both at childhood and a young adult age had a nearly fourfold increased risk of adult type 2 diabetes compared with men that were never overweight. The observational nature of our study precludes making conclusive statements about the observed associations, but our findings can be useful for hypothesis generation. If our findings were later found to be causal, it would indicate that overweight boys might reduce their risk of adult type 2 diabetes if they normalize their BMI during puberty. Our observation that boys that were normal weight at childhood and developed overweight during puberty had a fourfold increased risk of adult type 2 diabetes compared with boys with persistent normal weight supports the notion that BMI change during puberty is an important determinant of adult risk of type 2 diabetes. Subjects with early onset type 2 diabetes are more prone to macrovascular complications such as myocardial infarction and stroke compared with subjects with late-onset type 2 diabetes (28). This notion together with our present finding that a high BMI increase during puberty is more strongly associated with increased risk of early compared with late-onset type 2 diabetes indicate that our previously observed associations between a high BMI increase during puberty and increased risk of cardiovascular diseases in the BEST cohort are at least partly mediated via increased risk of early onset type 2 diabetes (16–18).

Obesity is central to the development of insulin resistance and type 2 diabetes (29, 30). It has been suggested that adipose tissue expansion, especially in visceral adipose depots, triggers low-grade inflammation associated with insulin resistance (29–33). In a previous study, using a subsample of the present cohort, we found that a high BMI increase during puberty was associated with substantially increased visceral fat mass (19), and we speculate that a high BMI increase during puberty might result in increased risk of type 2 diabetes via expansion of visceral fat mass, resulting in low-grade inflammation and insulin resistance. An effect of high childhood BMI on insulin sensitivity and future risk of adult type 2 diabetes is supported by a recent finding from the Bogalusa Heart Study showing that childhood obesity precedes hyperinsulinemia and the development of type 2 diabetes in adult life (34).

Recently, a Danish study reported that overweight at age 7 was associated with increased risk of adult type 2 diabetes if the overweight status was maintained until 13 years of age (35). These results are in concordance with the results from the current study, although it should be emphasized that BMI at age 13 is affected by both adiposity and pubertal timing and is therefore ambiguous. In contrast, BMI change during puberty, used in the current study, correlates only marginally with childhood BMI (16) and reflects the overall BMI change during the complete pubertal period. We, in this study, demonstrate that childhood BMI is a modest independent determinant, whereas BMI change during puberty is a substantial independent determinant of adult type 2 diabetes risk in men. Furthermore, and in line with the conclusions from the Danish study, our findings in the present Swedish cohort indicate that overweight boys might reduce their risk of adult type 2 diabetes if they normalize their BMI during puberty.

The limitations of the current study include that information on BMI later in life was not available. Without this information, it is not possible to determine definitely if the BMI data from childhood and during puberty add information beyond adult BMI for the prediction of adult type 2 diabetes risk. Moreover, we lack information on family history of diabetes and smoking. Another limitation is that type 2 diabetes diagnoses were captured through hospital-based registers, thus overlooking patients who were never treated at a hospital. However, the prevalence of a type 2 diabetes diagnosis was only slightly lower (4.9%) in the study population, corresponding rather well to the recently reported prevalence among Swedish men (36). The results may have limited generalizability to other ethnicities with a higher prevalence of type 2 diabetes, where Sweden, and northwestern Europe, rank very low (2). As Sweden did not have mandatory female military conscription, we were unable to retrieve young adult BMI for women, which made it impossible for us to determine sex-based differences in the associations between the developmental BMI parameters and risk of adult type 2 diabetes in women. The strengths of the study include that we used a population-based cohort including as many as 36,176 individuals representing a vast majority of the background population, with BMI available both before and shortly after puberty. Moreover, health care in Sweden is provided free of charge, which makes socioeconomic bias in diagnosis and representativeness unlikely.

In conclusion, BMI change during puberty is an important determinant of adult type 2 diabetes risk in men. These findings indicate that overweight boys might reduce their risk of adult type 2 diabetes if they normalize their BMI during puberty.

## Abbreviations:

BEST: BMI Epidemiology Study
BMI: body mass index
HR: hazard ratio
ICD: *International Classification of Diseases*
PIN: Personal Identity Number
SHC: School Health Care
